# Rabies and Strongylus Tetracanthus as a Coincidence in the Horse

**Published:** 1890-09

**Authors:** G. W. Butler


THE JOURNAL
OF
COMPARATIVE MEDICINE AND
VETERINARY ARCHIVES.

VOL. XI. SEPTEMBER, 1890. NO. 9.

### RABIES AND STRONGYLUS TETRACANTHUS AS A COINCIDENCE IN THE HORSE.

BY G. W. BUTLER, V.S.

IT is my intention to bring before the members of our association who are present to-day, a report of a few interesting cases that have come under my observation during the last few months.

CASE I.—On the evening of February 12th, 1890, I was called to see a trotting bred filly two years and nine months old, owned by John W. Brown & Son, of Yellowbud, Ohio. The history of the case, as obtained from the attendant, is as follows: Nothing unusual had been noticed with the animal until the morning of the day I was called, when it did not eat its food, after being taken from the yara where it had been the previous night and put into the stable. It stood stretched out in the stall, with its head hanging low and trembled violently, especially behind. There was a spasmodic contraction of the muscles, and the animal soon began to show symptoms of brain affection by placing its head against the manger and sides of the stall and pushing with great force. Trembling and spasms increased in severity until the animal was no longer able to retain the standing posture, when it fell down. When I arrived I found the following symptoms exhibited: position quiet and flat upon its side; respiration heavy and stertorous; pulse imperceptible; heart beating seventyfour per minute; passing urine involuntarily; limbs rigid and cold, and jaws set.

*Diagnosis.*—Some affection of the brain, probably a tumor or blood extravasation from a blow of some kind.

*Prognosis.*—Death, which took place two hours later.

*Post-mortem next morning.*—Body well nourished; small intestines filled with bloody-looking serum and the mucous membrane greatly inflamed; cæcum and large colon contained considerable fæcal matter, were greatly distended with gas, and the mucous membrane, which appeared congested and thickened, presented numerous red or dark colored spots, each of which contained one or two small round parasites (Strongylus Tetracanthus); other organs were healthy.

I failed to find any lesion of the brain, and I acknowledge my disappointment, for the symptoms presented certainly indicated an affection of that organ. I sent a few of the worms to Prof. Duncan, of Toronto, Canada, who pronounced them as belonging to that form of nematode parasite above mentioned.

CASE II.—A horse belonging to W. Leist, of Circleville, Ohio, became sick February 24th, 1890. He was a gelding, in fair condition and nearly four years old. The first symptoms noticed were spasmodic cramps, which seemed to be confined to the abdomen and were not unlike common spasmodic colic, although the animal appeared more nervous and excitable than in an ordinary case of colic. I was called on the 25th in the forenoon, when the horse exhibited the following symptoms: Pulse, 52 per minute; temperature, 101½° F.; pupils dilated; visible mucous membrane congested; abdomen gaunt; would strain often and violently, and at each time eject a little dark colored urine; passed a small quantity of fæces quite often; examination per rectum revealed the bladder empty and contracted, so that it felt like a small hard ball between the fingers. The horse would snap and bite at hens that happened to come near him, and any noise apparently incited a considerable degree of excitement, when the breathing would become hurried and parts of the body wet with perspiration. If one chanced to cough or spit near the horse he would strike out with the forefoot; the manner in which the act was performed, however, indicated that there was no desire on the animal's part to injure the offender, as the foot was raised and lowered in a moment of excitement and the blow did not seem to be directed at any particular object.[1] At times abdominal pain seemed severe in the extreme; and when we attempted to administer a drench contraction of the muscular system was so great that the horse could not stand and fell down. These spasms were clonic in nature, for as soon as the horse was left alone he would become comparatively quiet and partake of food and water, but seemed to have some difficulty in swallowing. The period of quietude between paroxysms varied as the disease advanced, becoming shorter all the time, and the spasms also grew more severe as death approached. The horse became very excitable before death, and died a few hours after I first saw him.

*Autopsy next morning.*—Abdomen greatly distended with gas; stomach, in addition to a quantity of food, contained quite a number of bots, but otherwise looked healthy; small intestines inflamed in places; mucous membraine of large colon and cæcum contained a great number of small worms (Strongylus Tetracanthus), and was thickened and inflamed. Thousands of small worms, nearly white, and from one quarter to one inch in length, were free in a portion of the large colon. The mucous membrane of the larynx, pharynx and trachea was inflamed and the lungs congested. As I had attempted to administer a dose of chloral in a pill which broke in the mouth, and as there was difficulty in swallowing, I thought at the time that the inflammation of the throat was probably due to the chloral.

Case III.—A mare two years and ten months old, rather small, owned by the same man who owned Case II, and kept on the same farm, was noticed unwell, March 9th, but had been slightly troubled with a cough for some time and discharged a little from the nose. I saw her March 11th, when I found pulse 44 per minute and full; temperature, 103⅔° F. Very nervous at times; would have cramps every few minutes; eyes bright and full, pupils widely dilated. In fact, she appeared as though very much frightened. The mucous membranes were injected, and there was great trembling; could not administer a drench on account of spasms; abdomen very gaunt; would eat during intervals between spasms; passed a little urine and fæces often, and there was considerable tenesmus. During paroxysms the respirations were very much accelerated, and the animal perspired at

[1] A point against diagnosis of rabies.—Ed.

times; would make frequent attempts to lie down before doing so, and would only lie a few minutes, when she would rise with great difficulty on account of muscular contraction.

March 12th. Pulse 64, irregular and varied, according to periods of quietude and excitement. The inclination to bite one of her fore legs was so great that it was necessary to muzzle her; spasms became more aggravated, and came on oftener; died toward evening, and before death was unable to rise to her feet.

*Autopsy*, March 13th at 2 P. M.—Abdomen distended with gas to such an extent that when the skin was cut the pressure from within was sufficient to tear the muscles; there was also an effusion of gas into the cellular tissue under the skin of the neck. The intestines and stomach contained some solid matter, and were inflamed in places; a great number of parasites (Strongylus Tetracanthus) were found in the mucous membrane of large colon and cæcum, and a few in floating colon. Those in floating colon were more scattered, and the spots on the mucous membrane where worms were found were much larger, being about the size of a pea and red in color. A great many small worms were found free in a portion of the large colon similar to Case II. The liver presented a number of light colored spots, and its parenchyma was soft, being easily broken down; numerous ecchymosed spots were scattered along the intestinal tract. There were a number of black spots on peritoneal coat of small intestines which resembled ink stains, and the mucous membrane immediately beneath these spots presented pit-like depressions, but there were no ulcers. The lymphatic glands along colon that contained parasites were enlarged, lungs congested, mucous membrane of larynx, pharynx and trachea inflamed, and brain congested.

*Remarks.*—In Case I the symptoms were such as to impress one with the expectation of finding some brain lesion, but, as stated above, the post-mortem revealed nothing unnatural in that organ. I cannot account for the symptoms of brain affection, as I think it scarcely probable that they were due to the irritation set up in the intestines by worms, and I think it equally improbable that an abnormality of the brain, such as a tumor or blood extravasation, would escape my notice in making a pretty careful examination. Judging from the amount of intestinal inflammation in Case I, I would think there must have been symptoms of abdominal pain exhibited in the early stage of the attack; and this may have been the case in the night, when the animal was not under observation. Cases II and III certainly presented symptoms of rabies, and my suspicion of the presence of that disease was at once aroused when I was first called to see Case II; yet a searching inquiry as to the manner in which the animal might have received the virus elicited nothing to make the matter clear; no one knew of there having been a rabid dog in the neighborhood. This animal had, during his illness, a small sore on the upper lip, but how he came by it, or what caused it, no one knew; and as I was the first to notice it, and as the horse had been sick some time before I saw him, I thought it quite probable at the time that it was caused by striking the head against something during one of the paroxysms of the disease, therefore I did not attach much weight to it. After holding a post-mortem and finding so many parasites and a good deal of inflammation produced by them, I rather attributed all the nervous excitement and muscular contraction to their presence. When I was called to see Case III, and found the symptoms almost identical with Case II, my suspicion of rabies was again aroused; but after making an examination of the animal after death, and finding so many worms again, and evidence of their injurious effect upon the intestines, I again nearly gave up the idea of rabies, and thought the trouble due to the parasites.

I have, however, recently obtained information that leads me to more fully conclude that Cases II and III suffered from rabies. Upon the farm where these horses were there were two dogs, one of which became sick a few days after the last horse died. The symptoms, as described by the owner of the animals, were: dropping of the lower jaw, a great desire to bite the ground, inability to control himself, and in a short time he was unable to get up, when he was destroyed. The dog made no attempt at any time, however, to bite those around him. Judging from this description, I have come to the conclusion that the dog had dumb rabies, and I have also come to the conclusion that in Cases II and III there was rather a strange coincidence of rabies and strongylus tetracanthus. Since these horses died I have seen four cases of dumb rabies in the dog in Circleville, and it has been reported that mad dogs were in the surrounding country.

I have never seen a case of rabies in the horse beside these which I have related, but taking into consideration the symptoms as described by different authors whose works I have had access to, the cases above mentioned are certainly somewhat different from those usually met with, as they did not manifest any great inclination to bite any one. The variation from a natural secretion of saliva was not sufficient to constitute any great abnormality, as there was no marked frothing at the mouth, neither did they become so furious as I should expect animals to that were suffering from rabies.

It may be that lesions in the intestines, caused by the parasites, had a tendency to somewhat modify the symptoms of rabies and render the cases apparently more complicated.

The most prominent symptom presented in these two cases of rabies was the clonic spasms, which seemed to be particularly severe in the muscular tissue about the abdomen. Perhaps this can be accounted for by the great number of parasites found in the colon and cæcum.

Cobbold, in his work on "Entozoa of Man and Animals," gives quite a detailed account of the different outbreaks caused by these parasites in Great Britain, as reported by different veterinarians and as studied by himself. He describes one case in which the symptoms somewhat resemble those manifested in the cases I have described.

Williams, in his "Treatise," states that the animals which suffered from these parasites in an outbreak in 1874 were all rising two years old, and that older animals running with them remained well and free from the parasites. He also states that, in his opinion, in conjunction with the ages of the affected animals, diarrhœa and emaciation may be considered as diagnostic symptoms. In the cases which I have described diarrhœa was absent, the animals were in fair condition, and in Case III the horse was nearly four years old.

The intestines in which parasites were found presented a nearly similar appearance to the descriptions given by Cobbold and Williams. The mucous membrane presented a great number of small brown or reddish spots, which contained the parasites. Some of these spots contained two worms and others only one, and they were either coiled in a round ring or in the shape of the letter S. The mucous membrane was inflamed and thickened, and in it were a few pus deposits, or small abscesses, in which the pus was usually inspissated.

Cases II and III, as well as other animals on the farm, had derived their drinking water sometimes from a well, at other times from an outlet of tiles that were put in the ground to drain a low part of the farm.

After the death of the two animals I prescribed ol. terebinth., alternated with ferri sulph. and gentian, for other horses on the farm, and all have done well.

I did not attempt a study of the growth and development of these parasites, neither did I perform any experiments in the way of feeding them to other animals.

In an interesting letter addressed to me not long since in reference to his experience with this parasite, Tait Butler, V.S., of Davenport, Iowa, says: "I was called to see a trotting-bred mare, 4 years old, that to me presented no other symptoms than those usually observed in those cases of indigestion or mal-assimilation so frequently met with in animals of that age. I prescribed quinine and strychnine, with little other effect than to cause the expulsion, along with the fæces, of a large number of small red worms of from a quarter to three-quarters of an inch in length. I immediately began the use of turpentine along with the tonics, but, owing to the advanced state of the case, the animal died. Several others of the same herd of horses were diseased but rapidly recovered under the treatment above indicated. The Winter following, the parasites again made their appearance, but with turpentine were again detroyed, never to appear again up to date. On the animal that died I held a post-mortem, and found the large intestines of a dark color, indicating a subacute inflammation of a very extensive nature. Instead of finding one or two black spots, indicating the abode of one or more parasites on a square inch of the mucous membrane, I found twenty. In fact, a careful estimate indicated that there were not less than seventy-five thousand of these parasites infesting the large intestines of this animal; and this notwithstanding that the animal had passed in the fæces during the two weeks preceding death an equally large number. In the intestines were found many of the larger white variety mentioned by Prof. Williams, varying in length from one to two inches.

"I first placed a number of the small red worms of, say from three-eighths to one-half an inch in length, in a temperature of twenty-eight degrees below zero, and kept them there for three days, when they were carefully thawed out and kept for twenty-four hours in well water at a temperature of 60°. During this twenty-four hours they increased at least 100 per cent. in length and lost much if not all of their red color. Some of these worms became at least an inch and a quarter long, and resembled in many respects the smaller white worms found in the intestines. The worms that were one and two inches long when taken from the intestines, grew to two and three inches long in water in which was mixed some ot the manure.

"While I did not succeed in growing a white worm of three inches in length from a red one of a quarter of an inch in length, I did, as above indicated, succeed in growing each specimen procured in such a manner as to lead me to think that probably the long white worm was only an advanced stage of the small red worm. In the mucous membrane were numerous small abscesses varying in size from a small pin head to half the size of a pea. In each of these there was a well-developed small red worm. Where these abscesses had appeared close together they frequently collapsed, and in this, which constituted the larger abscess, there were generally found two or three larger pale red worms.

"Some of these larger abscesses, however, only contained one worm, but in these cases it was generally from three-fourths to an inch in length and nearly white. I inferred from the above facts that the parasite escaped from the mucous membrane into the intestine only when it had produced this abscess and it had ruptured.

"I made sections of several specimens, but in even the most minute specks which appeared on the surface of the mucous membrane, I was able to find a completely formed worm, many of which were not more than one-twelfth of an inch in length. In no case was I able to find what might be construed into representing the ova of the parasite, or the parasite in its embryo stage. I was not able to find the parasite in any other part of the horse than the intestines, and portions of the large colon when fed to a kitten and two pups failed to produce the parasites in these animals, although they swallowed hundreds of them. The parasites always lost their bright red color when left to grow in water, and no degree of cold seemed sufficient to check their growth if they were kept in a temperature of not less than 60° after being thawed out.

"I may also state that these horses of which I speak were pastured on low land, and received their drinking water from a well three feet deep, and situated in the lowest part of the field. The water was dipped up with a pail into a trough.

"The owner of the horses, who is a reliable man of perhaps more than ordinary intelligence, informed me that when they removed the mud from the bottom of the trough, thousands of small red worms apparently identical with those passed by the horses were found in it."

